# Identification for the First Time of Cyclo(d-Pro-l-Leu) Produced by *Bacillus amyloliquefaciens* Y1 as a Nematocide for Control of *Meloidogyne incognita*

**DOI:** 10.3390/molecules22111839

**Published:** 2017-10-27

**Authors:** Qaiser Jamal, Jeong-Yong Cho, Jae-Hak Moon, Shahzad Munir, Muhammad Anees, Kil Yong Kim

**Affiliations:** 1 Division of Food Technology, Biotechnology and Agro chemistry, Institute of Environmentally-Friendly Agriculture, Chonnam National University, Gwangju 61186, Korea; qaiserjamal71@yahoo.com; 2 Department of Food Science and Technology, and Functional Food Research Center, Chonnam National University, Gwangju 61186, Korea; jyongcho@mokpo.ac.kr; 3 Department of Food Science and Technology, BK21 Plus Program, Chonnam National University, Gwangju 61186, Korea; nutrmoon@jnu.ac.kr; 4 Faculty of Plant Protection, Yunnan Agricultural University, Kunming 650201, Yunnan, China; shazid_10@yahoo.com; 5 Department of Microbiology, Kohat University of Science and Technology, Kohat 26000, Pakistan; muhamadanees@yahoo.com

**Keywords:** *Bacillus amyloliquefaciens* Y1, nematode, cyclo(d-Pro-l-Leu), second-stage juveniles

## Abstract

The aim of the current study was to describe the role and mechanism of *Bacillus amyloliquefaciens* Y1 against the root-knot nematode, *Meloidogyne incognita*, under in vitro and in vivo conditions. Initially, the exposure of the bacterial culture supernatant and crude extract of Y1 to *M. incognita* significantly inhibited the hatching of eggs and caused the mortality of second-stage juveniles (J2), with these inhibitory effects depending on the length of incubation time and concentration of the treatment. The dipeptide cyclo(d-Pro-l-Leu) was identified in *B. amyloliquefaciens* culture for the first time using chromatographic techniques and nuclear magnetic resonance (NMR ^1^H, ^13^C, H-H COSY, HSQC, and HMBC) and recognized to have nematocidal activity. Various concentrations of cyclo(d-Pro-l-Leu) were investigated for their effect on the hatching of eggs and J2 mortality. Moreover, the in vivo nematocidal activity of the Y1 strain was investigated by conducting pot experiments in which tomato plants were inoculated with *M. incognita.* Each and every pot was amended 50 mL of fertilizer media (F), or Y1 culture, or nematicide (N) (only once), or fertilizer media with N (FN) at 1, 2, 3, 4 and 5 weeks after transplantation. The results of the pot experiments demonstrated the antagonistic effect of *B. amyloliquefaciens* Y1 against *M. incognita* as it significantly decreases the count of eggs and galls per root of the tomato plant as well as the population of J2 in the soil. Besides, the investigation into the growth parameters, such as the length of shoot, shoot fresh and dry weights of the tomato plants, showed that they were significantly higher in the Y1 strain Y1-treated plants compared to F-, FN- and N-treated plants. Therefore, the biocontrol repertoire of this bacterium opens a new insight into the applications in crop pest control.

## 1. Introduction

A wide variety of economically significant crops is at high risk due to nematodes which are parasitic to plants and have a significant negative impact on various agricultural yields worldwide. Several species of plant-parasitic nematodes are known to cause serious reductions in yields of over 2000 vegetables, field crops, grasses and trees [1]. The root-knot nematodes, *Meloidogyne* spp., are considered as a significant problem in agricultural crop production [2]. *Meloidogyne* spp., are endoparasites, with a worldwide geographical distribution and broad host range [3]. They are a notorious reason behind substantial yield damage in the vegetable crops [4], including tomatoes, which are the most widely grown commercial crop, accounting for 14% of the world vegetable production [5]. Root-knot nematode infections weaken plants by disrupting their water and nutrient supply, which makes them more susceptible towards other opportunistic pathogens [6].

Root-knot nematodes may be managed by soil and chemical management practices and the selection of resistant varieties. The chemical management of root-knot nematodes has been preferred in conventional farming for increasing earnings, however, due to broad host range, short generation times, high reproductive rates, and endoparasitic habits, the control efficiency of root-knot nematodes by chemical nematicides is not fully effective. Moreover, these chemicals may cause serious human health effects and environmental contamination. Therefore, environmentally safe and economically feasible alternative control measures are always required. Effective antagonistic microorganisms are considered to be one of the potential alternatives to control root-knot nematodes [7] and extensive work has been conducted in this regard [8].

Microorganisms belonging to the genera *Bacillus*, *Paenibacillus*, *Pseudomonas*, *Streptomyces*, *Alcaligenes*, *Agrobacterium*, *Serratia*, *Clostridium*, and *Desulfovibrio* have been previously reported for their nematocidal activities [9]. The Gram positive, spore-forming *Bacillus* spp. are considered as biocontrol agents due to their capability to colonize the roots of plants and their long-term survival in the rhizospheric soil of plants [10]. They are also recognized as microbial factories for the production of diverse biologically active molecules. Some of these biologically active molecules are reported to have antibiotic properties [11,12] and most of them have been identified as low molecular weight dipeptides or cyclic peptides [13,14,15].

Several species of *Bacillus* are known to be able to be useful as biocontrol agents against root-knot nematodes including, *B. pumilus* [16], *B. megaterium* [17], *B. subtilis* [18,19], *B. firmus* [20], *B. thuringiensis* [21], *B. nematocida* [22], and *B. cereus* [23]. Their antifungal and antibacterial activities have made them a suitable choice for plant protection [24,25]. Application of *B. amyloliquefaciens* strain GB99 also promoted plant growth [26]. *B. amyloliquefaciens* are abundant in soil and are considered environmentally safe for application as biocontrol [12,27].

In our previous study, various antifungal metabolites were isolated from culture filtrate of a *B. amyloliquefaciens* Y1 strain that showed antagonism against some important plant pathogens [28]. Here, we evaluated the nematocidal effect of *B. amyloliquefaciens* Y1 against *M. incognita* under both in vitro and in vivo conditions and identified a nematocidal compound among its secondary metabolites to unveil its underlying antagonistic mechanism.

## 2. Results

### 2.1. Effect of BCS on Hatching of Eggs and Mortality of J2 of M. incogita

**Figure 1:**
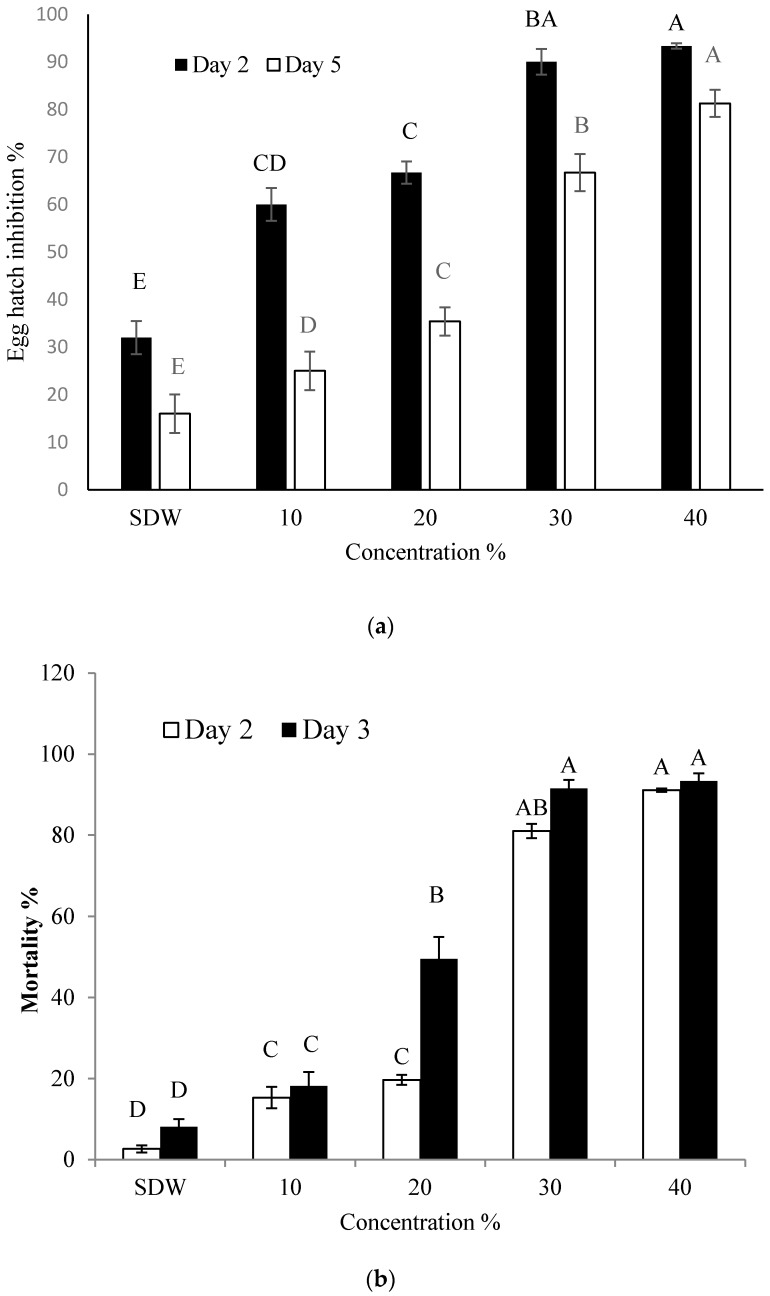
Effect of different concentrations of culture supernatant of strain Y1 on egg hatching (**a**) and mortality (**b**) of *M. incognita* at 26 °C for 2 and 5 days (eggs) and 1 and 3 days (J2) incubation. Serial distill water (SDW) was used as control. Error bars represent standard error of the mean from three replicates. Means with the same letter in same observation time are not significantly different at *p* ≤ 0.05 when compared with LSD.

Under in vitro conditions, various concentrations of the Y1 strain BCS significantly inhibited egg hatching and caused a higher J2 mortality in contrast to the control. The decrease in the hatching and increase in J2 mortality was noted to depend on the BCS concentrations and the length of the incubation period (Figure 1a,b). For example, 10–40% BCS concentrations inhibited the hatching by 32.5–60.6% after five days of exposure. Similarly, at day three of incubation, BCS caused mortality ranging from 40 to 80% at concentrations of 20%, 30% and 40%. Moreover, a significant increase in J2 mortality was observed with an increase in incubation period and concentration.

### 2.2. Effect of Crude Extract on Hatching of Eggs and J2 Mortality of M. incognita

**Figure 2:**
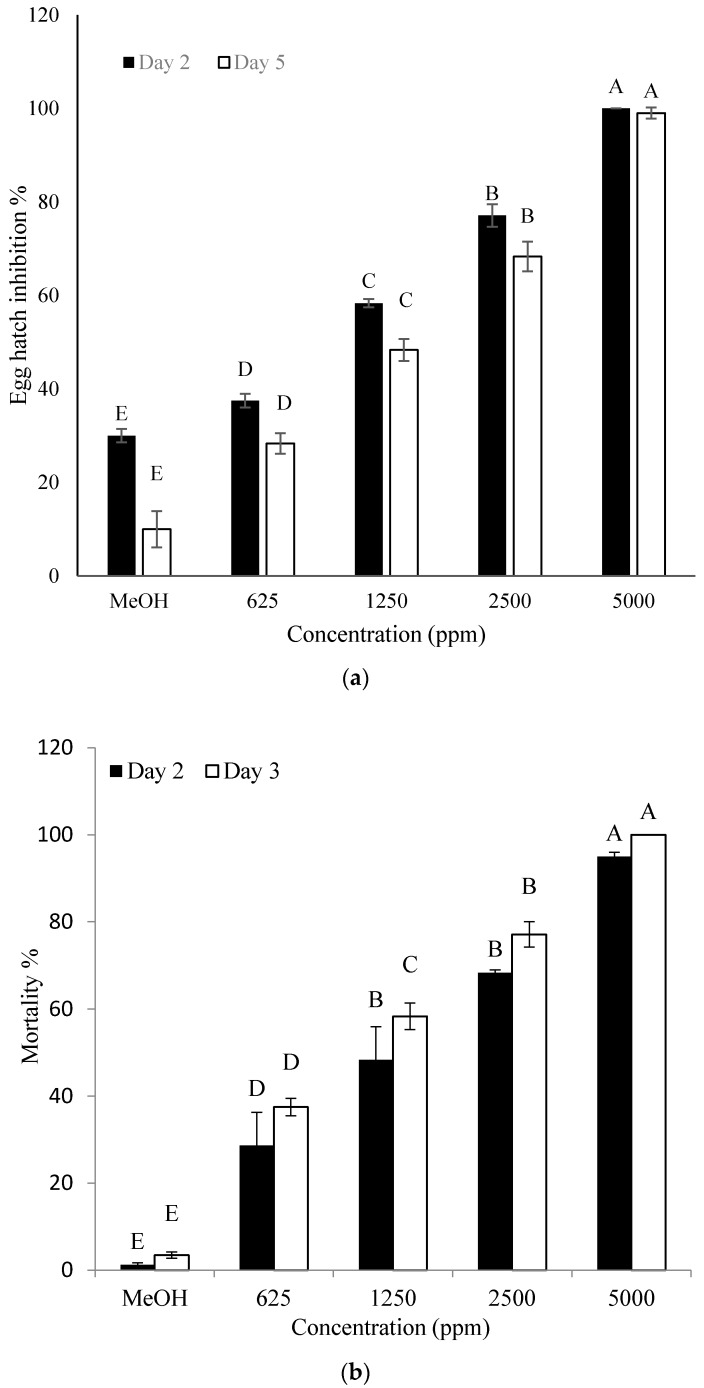
Effect of different concentrations of crude extract of strain Y1 on egg hatch (**a**) and second-stage juvenile (J2) mortality (**b**) of *M. incognita* at 26 °C for 2 and 5 days (eggs) and 1 and 3 days (J2) incubation. Methanol (MeOH) was used as control. Error bars represent standard error of the mean from three replicates. Means with the same letter in same observation time are not significantly different at *p* ≤ 0.05 when compared with LSD.

The *n*-butanol crude extract was found to have a significant effect on the hatching of eggs and J2 mortality. With the increase in crude extract concentrations and incubation time, a significant increase in hatch inhibition and J2 mortality was recorded. The crude extract significantly inhibited the hatching of eggs on the fifth day of incubation (Figure 2a,b). The inhibitory effect on the hatching of eggs was also observed even on the second day of incubation when higher doses (2500 and 5000 ppm) were used. When a high concentration of the crude extract was used against J2, mortality increased at both one and three days after incubation. More than 98% of J2 were dead as early as one day after incubation using 5000 ppm of crude extract.

### 2.3. Extraction and Purification of Nematocidal Compound

**Figure 3:**
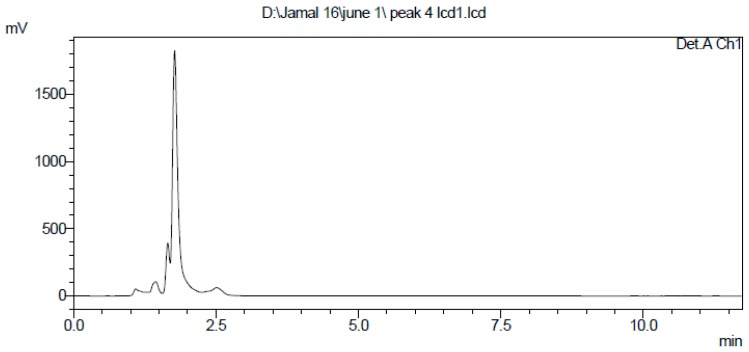
High performance liquid chromatography (HPLC) spectrum of the purified antifungal compound from *B. amyloliquefaciens* Y1.

The *n*-butanol crude extract of the BCS displayed nematocidal activity against *M. incognita*. Silica gel column chromatography yielded a bioactive fraction with significant nematocidal activity. That fraction was further purified by preparative HPLC and finally, 20 mg of a nematocidal compound was obtained. The purity of the compound was confirmed by a single peak at a retention time of 1.90 min using an analytical HPLC column (Figure 3).

### 2.4. Identification of the Purified Nematocidal Compound

**Figure 4:**
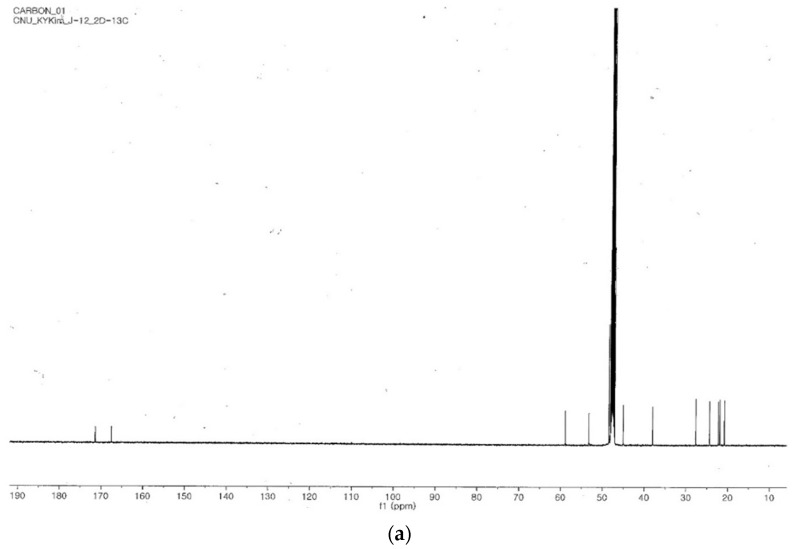

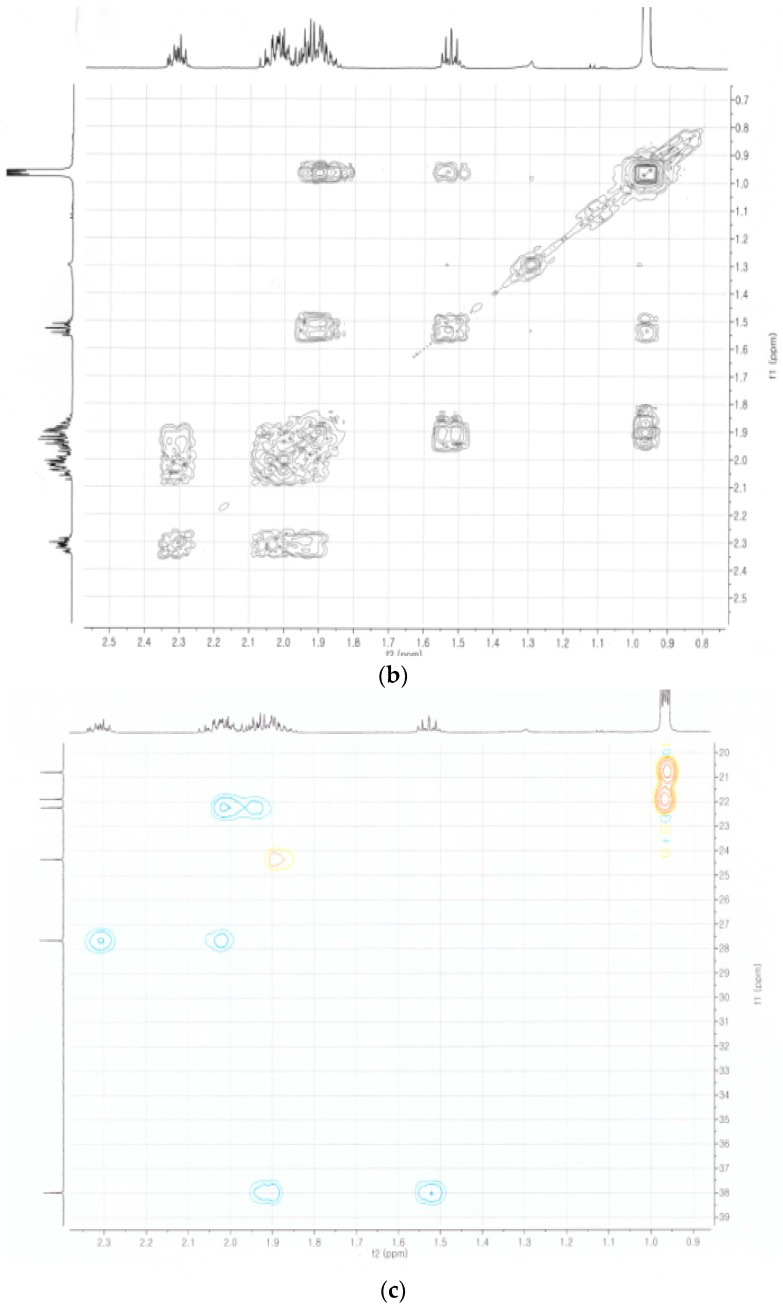

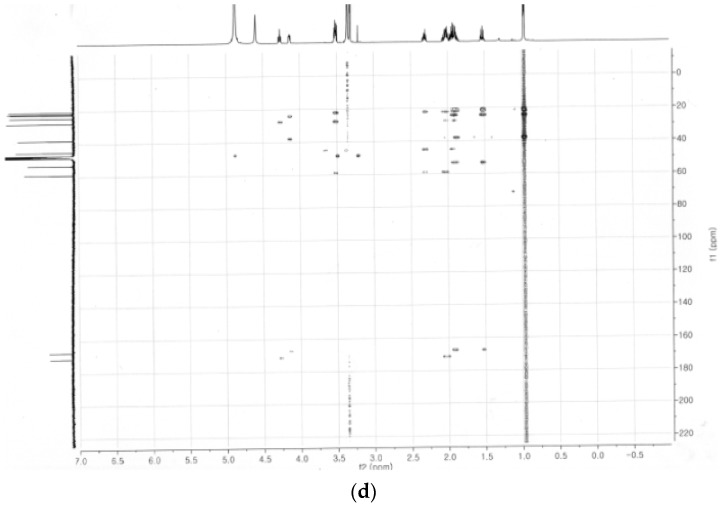
^13^C (**a**) 1H-1H COSY (**b**), HSQC (**c**) and HMBC (**d**) nuclear magnetic resonance (NMR) spectra of the purified compound from *B. amyloliquefaciens* Y1.

**Figure 5:**
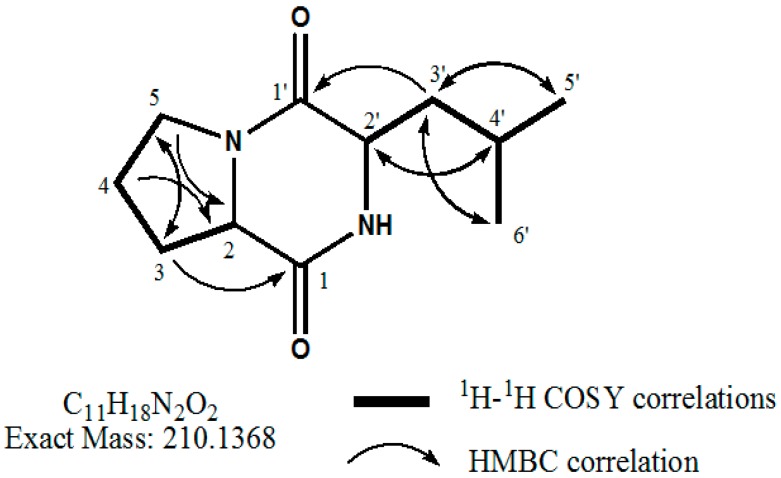
The structure of the purified compound from *B. amyloliquefaciens* Y1 cyclo(d-Pro-L-Leu).

The purified nematocidal compound was identified on the basis of its structure using ^1^H, ^13^C, 1H-1H COSY, HSQC and HMBC NMR spectra as shown in Figure 4a–d, respectively. The compound was thus identified as cyclo(d-Pro-L-Leu) and confirmed to be similar to the compound reported by Quyen et al. [29]. The proposed structure of cyclo(d-Pro-L-Leu) is shown in Figure 5.

### 2.5. Effect of the Identified Compound on Hatching of Eggs and J2 Mortality of M. incognita

**Figure 6:**
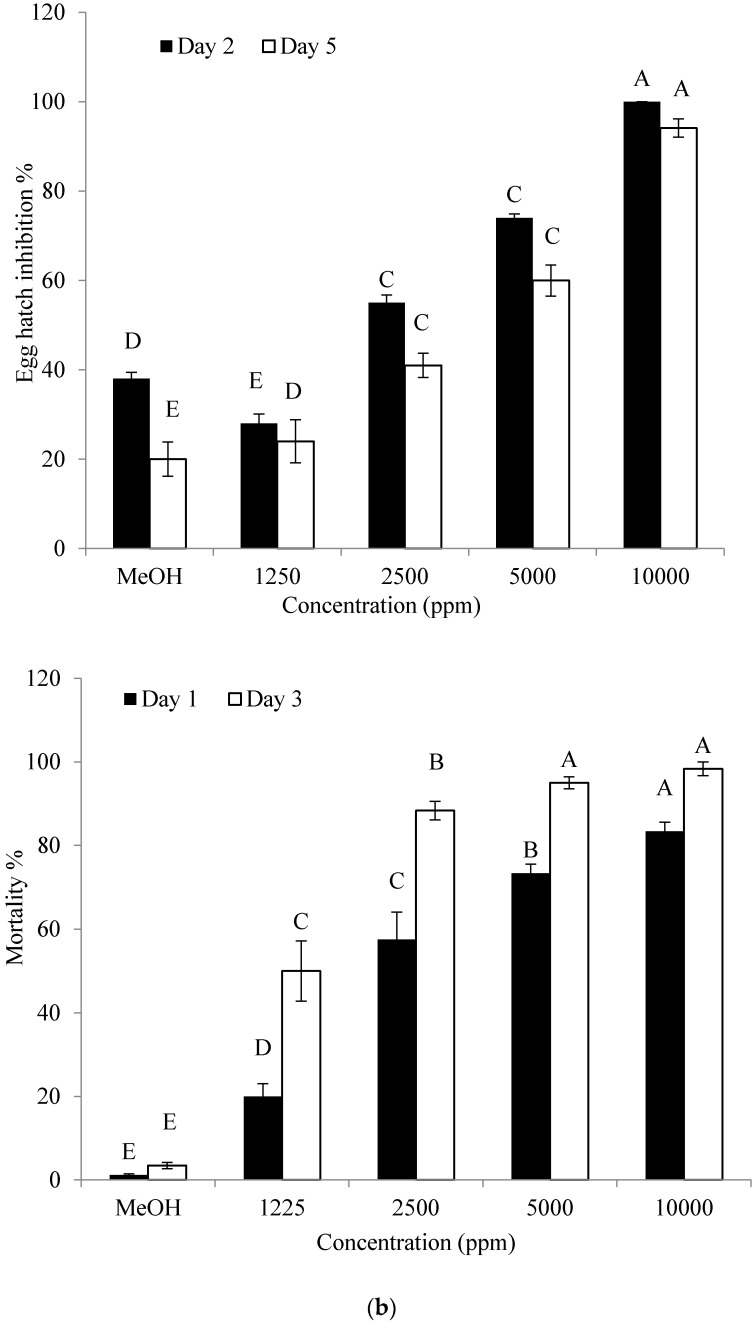
Effect of cyclo(d-Pro-l-Leu) purified from *B. amyloliquefaciens* Y1 culture on egg hatch (**a**) and second-stage juvenile (J2) mortality (**b**) of *M. incognita* at 26 °C for 2 and 5 days (eggs) and 1 and 3 days (J2) incubation. Methanol (MeOH) was used as control. Error bars represent standard error of the mean from three replicates. Means with the same letter in same observation time are not significantly different at *p* ≤ 0.05 when compared with LSD.

Cyclo(d-Pro-l-Leu) had a substantial effect on hatching rate and J2 mortality of *M. incognita*. Cyclo(d-Pro-l-Leu) significantly inhibited hatching of eggs on the 5th day of incubation (Figure 6a). The inhibitory effect on the hatching was observed even on the 2nd day after incubation when higher doses (5000 ppm and 10,000 ppm) were used. The J2 mortality also increased significantly with increase in dose of cyclo(d-Pro-l-Leu) on both the 1st and 3rd day of incubation. A substantial rise in J2 mortality was detected depending on the length of the incubation period and concentration (Figure 6b).

### 2.6. Effect of B. amyloliquefaciens Y1 on Incidence of M. incognita and Growth Promotion of Tomato in Pot Assays

**Figure 7:**
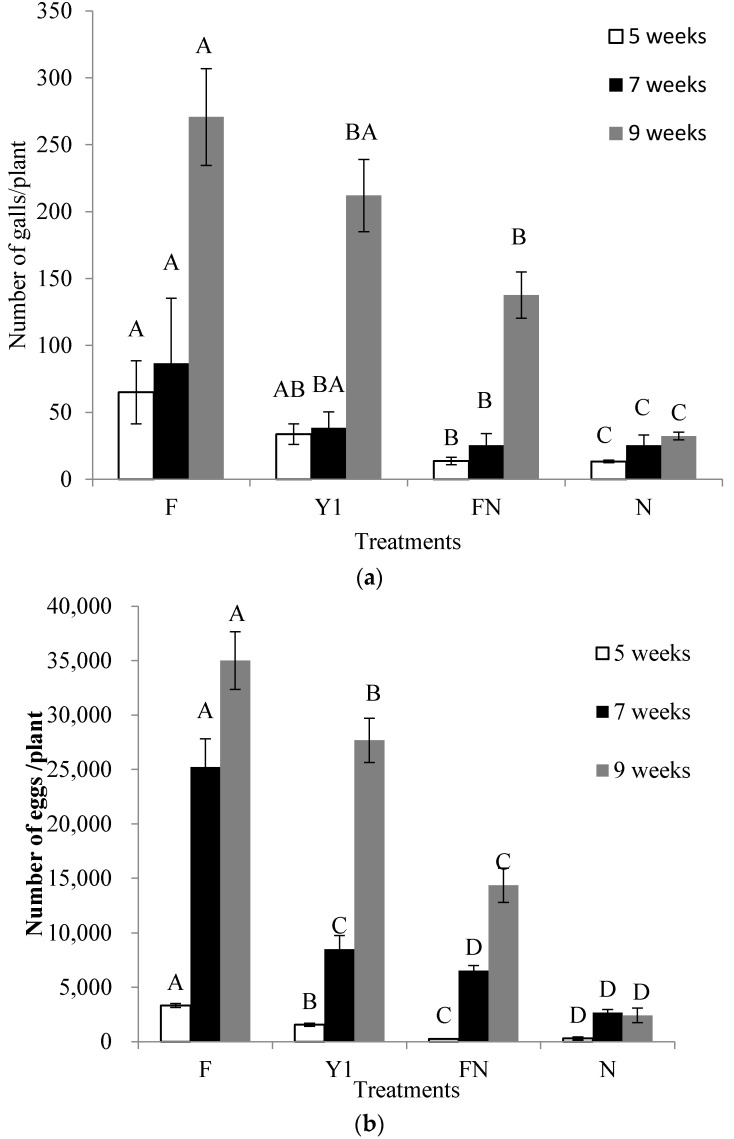
Effect of *B. amyloliquefaciens* Y1 on number of (**a**) galls and (**b**) eggs per tomato plants root treated with fertilizer medium (F), bacterial culture (Y1), fertilizer medium + commercial nematicide (FN) and commercial nematicide (N) at 5, 7 and 9 weeks after *M. incognita* infestation. Fertilizer medium was used as control and Mocap was used once as nematacide. Error bars represent standard error of the mean from four replicates. Means with the same letter in same observation time are not significantly different at *p* ≤ 0.05 when compared with LSD.

**Figure 8:**
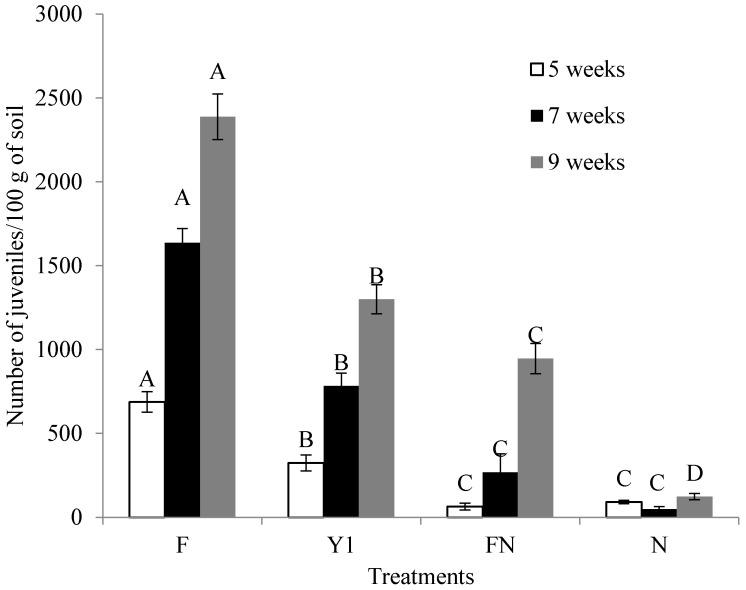
Effect of *B. amyloliquefaciens* Y1 on number of juveniles in 100 g soils in tomato plants treated with fertilizer medium (F), bacterial culture (Y1), fertilizer medium + commercial nematicide (FN) and commercial nematicide (N) at 5, 7 and 9 weeks after *M. incognita* infestation. Fertilizer medium was used as control and Mocap was used once as nematacide. Error bars represent standard error of the mean from four replicates. Means with the same letter in same observation time are not significantly different at *p* ≤ 0.05 when compared with LSD.

In pot experiments, the count of root galls, eggs and J2 mortality in the soil were recorded at 5, 7 and 9 weeks after *M. incognita* inoculation. These parameters increased from 5 to 9 weeks after infection with *M. incognita*. The chemical nematicide (N) and fertilizer with nematicide (FN) application were the most effective, followed by treatment with the Y1 strain (Figure 7a,b). Fertilizer-treated plants (control) had the highest rate of infection by *M. incognita*. In the tomato plants treated with nematicide, *M. incognita* caused low occurrences of root galling and exhibited a distinctly low population of J2 in soil. The count of galls and egg masses for each plant in Y1-treated plants at 5, 7 and 9 weeks after infestation with *M. incognita* showed a significant reduction compared to fertilizer-treated plants as well as lower J2 populations (Figure 8). Although FN and N treatments controlled nematodes effectively but these treatments were found to restrict the plant growth significantly. At the final sampling, the greatest fresh shoot weights were observed in plants treated by the Y1 strain (27.6 g) and were significantly higher than those by the F (21.6 g), FN (20.9 g) and N (15.1 g) treatments. The overall plant growth parameters, including shoot fresh weight, shoot dry weight and shoot length, increased significantly with application of Y1 strain (Table 1).

## 3. Discussion

In this study, *B. amyloliquefaciens* Y1 strain was tested for control of *M. incognita* both under in vitro and in vivo conditions. According to in vitro studies, the culture supernatant of Y1 strain showed nematocidal activity by inhibiting hatching of eggs and causing J2 mortality. The concentrations of the bacterial culture filtrate and incubation period directly affected the ovicidal and nematocidal activity. Mendoza et al. described that the culture supernatant of *B. firmus* had caused J2 mortality and inhibition of *M. incoginta* egg hatching [20]. Moreover, in our investigation, the *n*-butanol crude extract from Y1 strain had the ovicidal activity with more than 80% inhibition. In another similar finding, the ovicidal activity of *B. subtilis* crude extract on egg hatching was reported by Kavitha et al. [30]. The crude extract’s nematocidal activity in the present study was similar to that of the ethyl acetate and hexane crude extracts of *Pseudomonas lilacinu* and *P. aeruginosa* observed elsewhere [18]. In our previous study, Lee et al. reported that the culture filtrate and crude extract of *Lysobacter antibioticus* strain HS124 inhibited the hatching of egg and caused J2 mortality of *M. incognita* [31].

We isolated a namatocidal compound from the crude extract of Y1. We purified and identified cyclo(D-Pro-L-Leu) as the active compound through various chromatographic techniques and NMR analyses (^1^H, ^13^C, H-H COSY, HSQC, and HMBC). Cyclo(d-Pro-l-Leu) is a diketopiperazine [32]. Diketopiperazines (DKPs) are the smallest cyclic peptides, resulting from the head to tail folding of linear dipeptides and have an extensive range of biological properties, which include antibacterial, antifungal and antiviral effects [33]. Cyclo(Pro-Leu) was previously purified and identified from *Achromobacter xylosoxidans*, *Pseudomonas putida*, *Lactobacillus plantarum* and *Strepomyces* sp. by Degrassi et al. [34], Rhee [35], Yan et al. [36] and Dal et al. [37], respectively.

Ovicidal and namatocidal properties of DKPs against *M. incognita* have never been reported, so we consider our self as pioneers in reporting the role against root-knot nematodes of the DKP cyclo(d-Pro-l-Leu) taken from the strain Y1. Kumar et al. reported its antifungal properties, which allow it to stop the growth of *A. flavus* and *A. niger* on soybeans [38]. The crude extract from the Y1 strain may contain more than one type of active compounds because the *n*-butanol crude extract had greater inhibitory effect than the cyclo(d-Pro-l-Leu) alone on the hatching of eggs and J2 mortality. The same strain produced cyclo(d-Pro-l-Val) in our previous study, which showed antifungal activity against *F. graminearum* [39]. Benitez et al. reported that *B. amyloliquefaciens* LBM5006 produced a mixture of antimicrobial peptides in its culture broth [40]. *B. subtilis* and *B. amyloliquefaciens* were observed to possess biocontrol activity against nematodes having plant parasitic properties [41]. Several antimicrobial compounds were isolated from various strains of *B. amyloliquefaciens* to subdue the in vitro bacterial and fungal growth [42,43].

Our in vitro studies clearly showed that the Y1 strain had the potential to inhibit hatching of *M. incognita* eggs and to control J2 population by producing DKP-like cyclo(d-Pro-l-Leu) along with other biochemical active compounds. In the pot experiment, the Y1 inoculation suppressed infection caused by *M. incognita*, which caused a significant decrease in the root galls and count of eggs in the plant root system. Similar results were reported by Lee and Kim, who used *B. pumilus* L1 to control the root knot nematode in the pot experiment [16]. *B. pumilus* (ToIr-MA) was reported in another study to reduce the count of eggs and root galls in contrast to controls and promoted the plant growth [44].

Results from our in vivo experiments were consistent with the in vitro biocontrol effect of Y1 strain against hatching of eggs and increasing J2 mortality. Insunza et al. also stated that *B. cereus* was associated with the biocontrol of nematodes and promotion of plant growth [45]. According to the reports of Siddiqui and Mahmood, rhizobacteria are involved in controlling the populations of nematodes by several mechanisms, including production of antibiotics or enzymes, toxins, competition and parasitism as well as the induction of systemic resistance in plants [9]. Kong et al. reported that the production of various natural products such as cyclic peptidesvby *B. licheniformis* N1 strain that were responsible for the control of tomato grey mould, tomato late blight and pepper anthracnose [46]. Many bacteria involved in promoting plants’ growth accomplish this task by producing biochemically active compounds [47]. Similarly, our in vitro study demonstrated that the cyclic peptide cyclo(d-Pro-l-Leu) produced by Y1 acted as an antibiotic to suppress growth of *M. incognita* in plant root and soil. Thus, the production of cyclo(d-Pro-l-Leu) by Y1 in its culture broth may partially explain its biocontrol mechanism in the tomato pot experiments. In conclusion, we have confirmed the biocontrol potential of *B. amyloliquefaciens* Y1 against *M. incognita* causing disease in tomato plants. We also reported the ability of the strain to promote plant growth in the present study. This is the first report of the nematocidal effect of dipeptide cyclo(d-Pro-l-Leu) produced by Y1 strain, which is antagonistic against *M. incognita*.

## 4. Materials and Methods

### 4.1. Test Microorganisms

*Bacillus amyloliquefaciens* Y1 (accession no. KJ616752 in GenBank) was isolated from field soil collected at Chonnam National University (35.1764° N, 126.9081° E, Gwangju, Korea) and identified in our previous study. The pure culture of this strain was preserved in 25% glycerol solution at −70 °C.

### 4.2. Nematode Inoculum

Roots of the tomato plants (*Solanum lycopersium*) infested with *M. incognita* were collected from a local tomato field in Gwangju, South Korea and identified on the basis of morphology. Eggs of *M. incognita* were extracted from galled roots of tomato using 0.5% sodium hypochlorite solution and then rinsed with sterile water using sieves with 45-µm and 25-µm pores. The modified Baermann funnel method was used to incubate the eggs for 3–5 days to obtain second-stage juveniles (J2) [16]. *M. incognita* J2 were surface-sterilized with 0.01% streptomycin sulfate for an hour before use. The density of eggs and/or J2/mL of suspension was measured with five replicates.

### 4.3. Effect of Bacterial Culture Supernatant (BCS) on Hatching of Eggs and J2 Mortality of M. incognita

Luria-Bertani (LB) broth medium was used to grow Y1 at 40 °C for 7 days at 170 rpm. To prepare bacterial culture supernatant, the Lee et al. method was used [16]. After this, the culture broth was centrifuged for 20 min at 7000× *g*. The supernatant collected through centrifugation was then filtered successively by number 2 Whatman filter paper, using 0.45-μm and lastly 0.20-μm syringe filters. The 50 µL of nematode suspension containing approximately 200 eggs/80 J2 was poured in each of the 96 well-microtiter plates. The culture supernatant (0, 10, 20, 30 and 40 µL) was added to each well. Each well was filled with sterile distilled water (SDW) to make up a total volume of 100 µL suspension. The 0% BCS were considered as a control. After incubating at 26 °C, the figure of hatched juveniles was calculated at 2nd and 5th day of incubation, while J2 mortality was counted by determining the count of dead J2 at 1st and 3rd day of incubation, with the help of a stereoscopic microscope (SZX16, Olympus, Tokyo, Japan) at 50× magnification. Nematodes were thought to be dead if they did not show any movement when they were probed with the help of a fine plastic needle. All experiments were performed three times with three replicates.

### 4.4. Effect of Crude Extract on Hatching of Eggs and J2 Mortality of M.incognita

The pH of BCS (pH 6.4) was adjusted to a pH of 3 by using concentrated HCl. The crude extract was prepared using the Sophareth et al. method with some modifications [47]. Then the supernatant was extracted with *n*-butanol (1:1, *v*/*v*) to obtain the crude extract. The resultant extract was then concentrated by a rotary evaporator (Büchi, Rheinstetten, Germany) and evaluated for nematocidal activity. A 20% stock solution of the *n*-butanol crude extract was prepared. The effect of crude extract on hatching of eggs and J2 mortality was evaluated by treating 50 μL of nematode suspension containing 100 eggs/40 J2 approximately in the 96-well microtiter plate with 625, 1250, 2500 and 5000 ppm of the crude extract. Methanol was added at a final concentration of 5000 ppm, which was used as a control. The count of hatched juveniles from eggs was determined at the 2nd and 5th day of incubation, whereas J2 mortality was assessed by determining the count of dead J2 at 1st and 3rd day of incubation using an Olympus SZX16 stereoscopic microscope at 50× magnification after incubation at 26 °C. Nematodes were considered to be dead when they did not show any movement after probing them with a fine plastic needle. All experiments were performed three times with three replicates.

### 4.5. Extraction and Purification of the Nematocidal Compound

To purify the nematocidal compound, our previous study method to purify antifungal compound was followed with some modification [39]. The crude extract from the Y1 strain was mixed in methanol before silica gel column chromatography was performed (Kieselgel 60, 70–230 mesh, Merck, Darmstadt, Germany) with a stepwise elution of CH_3_Cl:MeOH (100:0, 90:10, 70:30, 40:60, 50:50, and 0:100; *v*/*v*). Fractions obtained after elution were concentrated in a vacuum (EYELA rotary vacuum evaporator, Bohemia, NY, USA) in order to obtain a semisolid mass. The active fraction was then subjected to high-performance liquid chromatography (HPLC) (SCL 10 A VP, Shimadzu, Kyoto, Japan) with a PrepHT C18 column (7 × 300 mm, 10 µm). The elution was monitored by using an SPD-10 A UV-VIS detector (Shimadzu, Kyoto, Japan) at 210 and 254 nm wavelength with manual injection. Every peak was distinctly obtained with help of acetonitrile and water as a mobile phase (35:65) at a flow rate of 2 mL/min. following this, all the peak fractions were concentrated using a centrifugal evaporator at 40 °C. Afterward, the purity of all obtained fractions was analysed further with the help of the HPLC analytical C18 column (2 µL, 4.6 × 250 mm). The resulting pure compound with a single peak was tested for nematocidal activity and used for further structural analysis.

### 4.6. Identification of the Purified Nematocidal Compound

Nuclear magnetic resonance (NMR ^1^H, ^13^C, H-H COSY, HSQC, and HMBC) was used to determine the purified compound’s structure. The purified nematocidal compound was dissolved in 0.6 mL methanol-*d*_4_ (CD_3_OD) before spectral analysis was conducted. NMR spectra were recorded using a DRX 500 NMR instrument (Bruker, Rheinstetten, Germany) working at 500 MHz for ^1^H, and 125 MHz ^13^C at 25 °C. Chemical shifts are stated in ppm (δ) and tetramethylsilane (CH_3_)_4_Si was used as an internal standard. ^1^H-NMR (ppm): 0.95 (3H, d, 6.6 Hz), 0.67 (3H, d, 6.5 Hz), 1.53 (1H, m, 3′b), 1.90 (1H, m, H-3′a), 1.88 (1H, m, H-4′), 4.13 (1H, m, H-2′), 2.01 (1H, m, H-4a), 1.95 (1H, m, H-4b), 3.52 (2H, m, H-5), 4.27 (1H, dt, 9.3 Hz, 1.5 Hz, H-2), 2.30 (1H, m, H-3a), 2.04 (1H, m, H-3b). ^13^C-NMR (ppm): 22.2 (C-6′), 23.3 (C-5′), 23.6 (C-4), 25.8 (C-4′), 29.1 (C-3), 39.4 (C-3′), 46.4 (C-5), 54.6 (C-2′), 60.3 (C-2), 168.9 (C-1′) and 172.8 (C-1).

### 4.7. Effect of the Identified Compound on Hatching of Eggs and J2 Mortality of M. incognita

The influence of the purified compound on the hatching of eggs and juvenile mortality of *M. incognita* was examined in a 96-well microtiter plate containing 100 eggs or 40 J2 per 50 μL of nematode suspension. Various concentrations of the purified compound dissolved in methanol were added to each well to make the final concentrations of 1250, 2500, 5000 and 10,000 ppm. For the control treatment, methanol was added to make a final concentration of 10,000 ppm. During the 5 days of incubation, the count of hatched juveniles from eggs was determined at the 2nd and 5th days, while J2 mortality was assessed by calculating the count of dead J2 at the 1st and 3rd days of incubation using an Olympus SZX16 stereoscopic microscope at 50× magnification. Nematodes were considered dead if they did not move when probed with a fine plastic needle. The experiment was performed with three replications.

### 4.8. Effect of B. amyloliquefaciens Y1 on Incidence of M. incognita and Growth Promotion of Tomato in Pot Assays

*Solanum lycopersicum* (tomato) seeds were sown in bed soil (Bio-bed soil Ι, Heong Nong Seed Co., Namyang, Korea) in 54 × 45 mm plastic cell plug trays. Six weeks after sowing, the tomato seedlings were transferred into (30 × 15 cm) pots containing 700 g of a combination of soil, sand and vermiculite (2:1:1, *v*:*v*:*v*) and kept at room temperature (28 °C) in an artificially lit up room (12,000 lux) for 16 h/24 h. Each pot was provided with 50 mL of fertilizer media (F) (KH_2_PO_4_ 0.08 g, KCl (0-0-60) 0.02 g, K_2_SO_4_ 0.1 g, CaCl_2_ 0.1 g, water soluble fertilizer 2.66 mL, blue fertilizer (20-20-20-2, 4 g) per litre of water); or bacterial culture (Y1) grown for 7 days at 40 °C in media known as BB media (chitin 0.5 g, gelatin 0.5 g, Yeast 0.1 g, KH_2_PO_4_ 0.08 g, KCl (0-0-60) 0.02 g, K_2_SO_4_ 0.1 g, CaCl_2_ 0.1 g, water soluble fertilizer 2.66 mL, blue fertilizer (20-20-20-2, 4 g ) and sugar 4 g per litre of water); or fertilizer media with nematicide (FN); or nematicide (N) alone (2.5 g l−1 Mocap, Bioscience of Korea, South Korea) at 1, 2, 3, 4 and 5 weeks of transplantation. Treatment provided only with fertilizer (F) was used as a control. The nematicide was applied only once at 1 week after transplanting. One week after transplanting, the tomato plants were infested with 4 mL of *M. incognita* suspension containing 10,000 eggs and 500 juveniles into two deep holes in the tomato rhizosphere. At 5, 7 and 9 weeks after *M. incognita* inoculation, tomato plants were removed from pots and then washed in tap water and results were recorded. The count of galls and eggs per root system of the plant was determined. A modified Baermann funnel technique was used to calculate the population density of J2 in the rhizosphere [30]. The number of eggs and J2 were counted under the stereoscopic microscope at 50× magnification. In every sampling period, shoot length and weight were recorded. This experiment was planned to have a completely randomized design (CRD) by using four treatments and six pots for every time period. The experiment was repeated two times.

### 4.9. Statistical Analysis

All the data were analysed by Statistical Analysis System 9.4 (2016, SAS Institute, Cary, NC, USA). The mean values were compared by the least significant difference (LSD) test at *p* ≤ 0.05 and presented as the mean values ± standard error.

## Figures and Tables

**Table 1:** Changes in plant height, fresh root and shoot weight as infected with infested with *Meloidogyne incognita.*

Weeks after NI	Treatments	Plant Height (cm)	Shoot Fresh Weight (g)	Shoot Dry Weight (g)
5	F	30.3 ± 0.8 b	13.3 ± 0.9 bc	1.5 ± 0.2 ba
	Y1	35.6 ± 0.8 a	17.2 ± 1.1 ba	1.8 ± 0.1 ba
	FN	35 ± 0.5 a	18.6 ± 1.6 a	2.1 ±0.1 a
	N	28.6 ± 1.8 ba	11.6 ± 1.8 c	1.3 ± 0.3 b
7	F	34.1 ± 2.1 ba	16.2 ± 1.4 bc	1.4 ± 0.1 c
	Y1	37.6 ± 0.3 a	21.3 ± 2.4 a	2.5 ± 0.1 a
	FN	33.3 ± 1.2 ba	17.4 ± 2.1 ba	2.1 ± 0.1 b
	N	28.6 ± 2.1 b	13.4 ± 1.8 c	1.3 ± 0.5 c
9	F	36.6 ± 1.2 ba	21.6 ± 1.7 b	2.4 ± 0.1 b
	Y1	40.1 ± 1 a	27.6 ± 1.3 a	3.2 ± 0.1 a
	FN	35.3 ± 1.5 b	20.9 ± 2.1 b	2.4 ± 0.1 b
	N	34.3 ± 1.2 b	15.1 ± 0.5 c	1.3 ± 0.2 c

NI = Nematode infestation; F = Fertilizer medium; Y1 = *Bacillus amyloliquefaciens* Y1; FN = Fertilizer + Nematicide; N = Nematicide. Fertilizer medium was used as control and Mocap was used once as nematacide. Calculated mean values are from four replicates and ± show standard error of the mean. Means with the same letter in the same observation time are not significantly different at *p* ≤ 0.05 when compared with LSD.
